# From design to implementation: policy evolution of home-based primary care in Korea

**DOI:** 10.1093/geroni/igag084

**Published:** 2026-07-21

**Authors:** Eunhee Choi, Jihwan Lee, Bokyoung Choi, Yumi Shin, Jihee Choi, Jakyung Lee, Ahreum Choi, Soong-nang Jang, Hyejin Lee, Chang-O Kim

**Affiliations:** Institute for Community Care and Health Equity, Chung-Ang University, Seoul, Republic of Korea; Department of Nursing, Graduate School, Chung-Ang University, Seoul, Republic of Korea; Institute for Community Care and Health Equity, Chung-Ang University, Seoul, Republic of Korea; Department of Child and Family Welfare, Daegu University, Gyeongsan, Republic of Korea; Institute for Community Care and Health Equity, Chung-Ang University, Seoul, Republic of Korea; Institute for Community Care and Health Equity, Chung-Ang University, Seoul, Republic of Korea; Graduate School of Public Health, Seoul National University, Seoul, Republic of Korea; Institute for Community Care and Health Equity, Chung-Ang University, Seoul, Republic of Korea; Red Cross College of Nursing, Chung-Ang University, Seoul, Republic of Korea; Department of Family Medicine, Seoul National University Bundang Hospital, Seongnam, Gyeonggi-Do, Republic of Korea; Department of Family Medicine, Seoul National University School of Medicine, Seoul, Republic of Korea; Institute for Community Care and Health Equity, Chung-Ang University, Seoul, Republic of Korea; Dolbom Clinic, Seoul, Republic of Korea

**Keywords:** Home-based medical care, Home care services, Homebound persons, Integrated care, Community health services

## Abstract

**Background and Objectives:**

South Korea’s rapid expansion of the older adult population, along with growing health and social care needs, has created an urgent demand for Home-Based Primary Care (HBPC). This study identified key design priorities for a Korean HBPC model and examined their reflection in the national pilot.

**Research Design and Methods:**

This study was designed as a case study of the development and implementation of Korea’s HBPC pilot program, using a dual-method approach for descriptive analysis. First, semi-structured interviews with 42 key informants, including policy makers, legal experts, researchers, and clinicians, identified design priorities using the 5P framework (Purpose, Population, People, Process, and Patterns). Second, policy documents and implementation guidelines from Phases 1–4 were reviewed to trace the program’s evolution.

**Results:**

Stakeholders emphasized that the primary objectives of HBPC should be to support aging in place, preserve functional ability, and reduce avoidable medical expenditures. Stakeholders supported gradually expanding the target population. However, a central point of contention involved whether to lower provider eligibility thresholds to increase scalability or raise them to ensure service quality. Document analysis revealed that revisions across Phases 1–4 expanded eligibility for recipients and providers and reduced coinsurance rates, thereby improving accessibility. Nonetheless, interdisciplinary training and a comprehensive performance evaluation framework remain in early stages.

**Discussion and Implications:**

Integrating stakeholder perspectives into policy design grounds HBPC development in practical experience. These findings underscore HBPC’s potential in supporting aging in place among high-need, vulnerable populations and inform the development of inclusive, adaptable community-based care models.

Innovation and Translational Significance: Rapid population aging and fragmented health and long-term care systems have created an urgent need for Home-Based Primary Care (HBPC) in South Korea. By integrating stakeholder interviews with longitudinal analysis of national pilot policies, this study clarifies how HBPC design priorities translate into real-world policy decisions. The findings show that adjustments in eligibility criteria, provider participation, and cost-sharing can meaningfully improve access for high-need, vulnerable older adults. These insights highlight HBPC’s potential to support aging in place and reduce avoidable institutional care, offering practical guidance for developing equitable and scalable community-based care models.

## Introduction

Home-Based Primary Care (HBPC) has emerged as a vital strategy for addressing the complex health needs of older adults, particularly those with multiple chronic conditions and functional impairments. By delivering medical services directly in patients’ homes, HBPC has been linked to improved continuity of care, fewer unnecessary hospitalizations, and enhanced experiences for both patients and caregivers, including higher satisfaction, better quality of life, and improved clinical outcomes (Nelson et al., 2014; Reid et al., 2010; Totten et al., 2016; World Health Organization, 2018). These features align closely with global health policy goals focused on improving outcomes for high-need, high-risk populations.

International models offer valuable insights into how HBPC can be designed and expanded to meet the needs of aging societies. In the United States, the Veterans Health Administration’s HBPC program, initiated in 1970, has consistently demonstrated reductions in hospitalizations and overall healthcare expenditures through comprehensive, multidisciplinary care delivered at home (U.S. Department of Veterans Affairs, n.d.; Edes et al., 2014; Cummings et al., 1990). The Independence at Home demonstration (2012–2023) evaluated the effectiveness of physician-led HBPC in reducing costs and enhancing care for high-need Medicare beneficiaries (Centers for Medicare & Medicaid Services [CMS], n.d.). Although its overall impact on spending and hospital utilization was inconclusive, the program demonstrated potential to improve access, care continuity, and responsiveness to patient needs (Kimmey et al., 2025). Another key model, Hospital-at-Home (HaH), was originally developed for the U.S. healthcare context and rigorously evaluated by a Johns Hopkins research team in the 1990s. HaH delivers hospital-level acute care in the home through daily visits, diagnostic testing, and continuous clinical oversight (Johns Hopkins Medicine, n.d.). Nationally expanded through the CMS Acute Hospital Care at Home waiver in 2020, HaH has been associated with lower mortality, reduced readmissions, decreased healthcare costs, and higher satisfaction among patients and caregivers, compared to conventional inpatient care (Caplan et al., 2012; Leff et al., 2024).

Japan operates a national health and long-term care insurance system that is administered in a decentralized manner by local municipalities. Physician home visits were included in national insurance coverage in 1986, establishing a formal policy foundation for home-based care (Kim et al., 2024). In 2006, simultaneous revisions to health and long-term care legislation led to the establishment of 24/7 Home Care Support Clinics (HCSCs), thereby strengthening the infrastructure for continuous in-home medical services. Japan’s home-based care model is structured around four core functions: discharge planning, routine in-home follow-up, emergency response, and end-of-life care (Nam, 2019). In 2012, the introduction of the enhanced HCSC designation aimed to further expand emergency and palliative care capabilities. Compared with standard HCSCs, enhanced HCSCs have been associated with higher rates of home-based end-of-life care and lower in-hospital mortality, suggesting improved capacity to fulfill patients’ preferences to remain at home at the end of life (Sun et al., 2023).

The need for HBPC in South Korea is particularly acute, as the country is aging more rapidly than any other member of the Organization for Economic Co-operation and Development (OECD). In 2021, 19.3% of the population was aged 65 years or older—slightly above the OECD average of 18.6%—and this figure is projected to reach 40.1% by 2050, the highest among OECD countries (World Bank, n.d.; OECD, 2023). The proportion of adults aged 80 and above is also increasing rapidly, rising from 3.9% in 2021 to a projected 16.5% by 2050, the fastest growth rate globally (OECD, 2023). Given that older adults frequently present with multiple chronic conditions and functional impairments, the expansion of integrated healthcare and long-term care services has become an urgent policy priority. Effective management of complex health conditions in this population requires a coordinated delivery system that integrates healthcare and long-term care to improve chronic disease management and reduce avoidable hospitalizations. South Korea has operated a universal, mandatory health insurance system since 1989, providing coverage to the entire population regardless of employment status (Ministry of the Interior and Safety, National Archives of Korea, n.d.). In addition to the National Health Insurance, which primarily finances physician- and hospital-delivered medical services, a separate Long-Term Care Insurance program was introduced in 2008 to support older adults requiring assistance with physical functioning and activities of daily living (National Health Insurance Service, n.d.). Although both schemes are administered by the National Health Insurance Service, they are financed and managed independently, contributing to systemic fragmentation between medical and long-term care services. Recent national data underscore the vulnerability of Long-Term Care Insurance beneficiaries. In the past year, 22.8% had at least one hospitalization, and the average time from initial eligibility determination to death was just 3.84 years (Lee et al., 2022; National Health Insurance Service, 2025). The need to navigate two separate coverage systems has resulted in substantial unmet medical needs among older adults, leading to avoidable healthcare utilization, premature institutionalization, and elevated mortality (Jones & Dolsten, 2024; Rahman et al., 2022).

Unlike many countries where home-based physician services have long been incorporated into primary care, Korea maintained strict legal restrictions on physicians providing medical care outside licensed medical institutions. As a result, physician-led HBPC remained underdeveloped until recent legislative and policy reforms established a legal foundation for its implementation. In 2018, the Korean government introduced a National Strategy for Community-Based Integrated Care to support aging in place by providing coordinated medical, social, and residential services within local communities (Ministry of Health and Welfare [MOHW], 2018). Later that year, an amendment to the National Health Insurance Act established the legal basis for physicians to deliver HBPC services (Lee, 2019). Building on this, the Ministry of Health and Welfare launched a pilot HBPC program in December 2022, designating existing clinics and hospitals to provide care to Long-Term Care Insurance beneficiaries through multidisciplinary home-visit teams comprising physicians, nurses, and social workers. The program operates under a mixed financing model, drawing on both National Health Insurance and Long-Term Care Insurance funds. This initiative represents a significant policy effort to address the longstanding fragmentation between medical and long-term care systems through an integrated, community-based primary care approach.

Overcoming these barriers require collaboration among policymakers and front-line professionals across multiple sectors. Engaging stakeholders is essential to ensure that HBPC models are contextually appropriate, operationally feasible, and sustainable within Korea’s unique health system and rapidly aging demographic context.

This study presents a policy and implementation case study of Korea’s HBPC pilot program, describing how stakeholder-informed priorities were incorporated into the development and evolution of the model across pilot phases. To this end, we employed a qualitative study design involving semi-structured interviews and document analysis of official program materials.

## Method

### Study design

This study employed a multi-source qualitative design comprising two components: (1) semi-structured interviews with stakeholders to identify priorities for HBPC model development; and (2) document analysis of government-issued materials, including recruitment notices, implementation plans, operational manuals, and press releases, related to Phases 1 through 4 of the HBPC pilot program, to track changes over time.

### Part 1. Stakeholder interviews to inform HBPC model design

#### Participants

Forty-two key informants were purposively selected based on their expertise in health policy, clinical medicine, public health, social welfare, and long-term care (Table 1). Data were collected through a combination of individual and group interviews, as well as written follow-up surveys. Nine individuals participated in one-on-one interviews, representing policy experts in long-term care and public health, as well as faculty in medicine, law, and nursing. Two focus groups were conducted: one in person with 16 health policy professionals and one online with seven clinicians. Additionally, 11 individuals contributed via written responses only. Four participants took part in both individual and group interview sessions.

**Table 1:** Data sources for the study.

Data source type	Subcategory/type	*N*	Notes/description
**Stakeholder interviews (*n *= 42)^a^**	Individual interviews		
	In-person	9	
	Written (email)	24	13 were follow-up interviews with previous participants (individual/group) 11 participated only via written responses
	Group interviews		
	Health policy professionals	19	In-person
	Clinical professionals	7	Online
**Policy documents (*n *= 38)^b^**	Public notices	8	Announcements for recruitment of HBPC pilot sites
	Implementation plans	3	Detailed implementation documents for HBPC pilot phases
	Guidelines	17	Operational manuals and standardized forms for HBPC service delivery
	Press releases	10	Official announcements on HBPC pilot expansion and program milestones

*Notes.* HBPC = home-based primary care.

a Due to overlapping participation between individual and group interviews, the total number of unique participants was 42.

b A complete list of documents, including publication dates and sources, is provided in Table S1.

**Table 2:** Demographic and professional characteristics of interview participants.

Characteristic	*n* (%)
**Gender**	
Male	28 (66.7)
Female	14 (33.3)
**Occupation**	
Policy researcher	12 (28.6)
Physician	16 (38.1)
Professor	14 (33.3)
**Academic discipline of professors**	
Medicine	11 (45.8)
Nursing	6 (25.0)
Social work	4 (16.7)
Public health	1 (4.2)
Law	1 (4.2)
Occupational therapy	1 (4.2)

*Notes.* Academic disciplines were categorized according to participants’ primary faculty affiliation at the time of the interview. Several individuals held overlapping academic or professional roles; therefore, the total number of disciplines exceeded the number of professors.

**Table 3:** Design considerations for an HBPC model in Korea: summary of stakeholder insights using the 5P framework.

Categories	Domain	Description
**Purpose**	Goals	Promote aging in place to ensure dignified living for older adults in their homes Maintain functional ability by managing chronic conditions and supporting healthy aging environments Reduce avoidable healthcare expenditures associated with population aging
	Operational priorities	Enhance accessibility for older adults with limited mobility Deliver integrated and continuous care services Strengthen linkages with the LTC system Prevent unnecessary hospitalizations and institutionalization Provide home-based end-of-life care
**Population**	Target population	Short-term focus: older adults with significant functional decline and limited access to care, particularly those classified as levels 1–5 under Korea’s LTC eligibility system Long-term focus Older adults at high risk of institutionalization Individuals transitioning from LTC facilities to the community Those requiring home-based palliative care Patients recovering from acute conditions Individuals needing short-term post-discharge support
	Referral pathways	Referral pathways may include: LTC institutions, local community service centers, hospitals involved in care transitions, and LTC hospitals
**People**	Administration	Types of implementing organizations: Designated centers: lower entry criteria enhance scalability; higher standards ensure quality Public sector models via local public health centers Geographic allocation tailored to regional characteristics Establishment of a central management and support authority
	Workforce	Core staffing Minimum team: physician, nurse, social worker Expanded team: minimum team plus nurse practitioner (NP), physical therapist (PT), occupational therapist (OT), and dietitian Flexible staffing standards based on local conditions Role substitution strategies for recruitment-constrained settings (e.g., NP or OT substituting where appropriate) Establishing a reimbursement system that supports workforce sustainability
	Training and capacity building	Specialized educational programs in universities and graduate schools Interprofessional education integrated into academic curricula Accredited training by professional societies Clinical education and training through university hospitals Ongoing training programs embedded in HBPC models Peer learning and capacity-building at the community level
**Process**	Core service components	Minimum standards (comprehensive, continuous care) Initial patient assessment and program enrollment Comprehensive geriatric assessment and individualized care planning Interdisciplinary case conferences Regular home visits for ongoing case management Family counseling and caregiver education Coordination of community-based resources (e.g., interagency meetings) Maximum service standards (to prevent institutionalization) include all of the above, plus: 24/7 emergency consultation and home visit availability Proactive home-based rehabilitation Support for inpatient stays and discharge transitions Home-based palliative and end-of-life care
	Supporting system components	Community resource integration: Community-integrated care consortiums Cross-sector service delivery incentives Shared data systems for patient information exchange Coordination with home nursing services: Integration of nursing functions within HBPC teams Collaboration between HBPC and external nursing agencies Payment and delivery model: Mixed funding from National Health Insurance and LTC Insurance Blended payment approach (fee-for-service and capitation)
**Patterns**	Performance indicators	Patient satisfaction, clinical effectiveness, healthcare utilization metrics, healthcare expenditure trends, and proportion of patients receiving home-based end-of-life care.

*Notes.* HBPC = home-based primary care; LTC = long-term care.

#### Data collection

Semi-structured interviews were conducted between April and December 2021 in Korean, using in-person, online, or email-based formats. Individual interviews lasted approximately 60 minutes, while group interviews extended to around two hours. All sessions were facilitated by the lead investigator (C.-O. Kim), with support from two research staff members (E. Choi and Ja. Lee).

The interview guide was developed based on Olsan’s clinical microecosystem framework, which includes five domains: purpose (program goals), patients (target population), process (service structure and delivery), people (interdisciplinary team), and patterns (outcome indicators and financial mechanisms) (Olsan et al., 2009). All interviews were audio-recorded with participant consent, transcribed verbatim, and supplemented by written responses. Ethical approval for the study was obtained from the appropriate institutional review boards.

#### Analysis

Template analysis was applied, using the five framework domains as initial coding categories. Subcategories were generated inductively (King, 1998). One researcher (E. Choi) conducted the coding using Atlas.ti software. Coding decisions and category refinement were reviewed collaboratively with the principal investigator (Kim) to ensure analytic consistency and methodological rigor. As this study was conducted as a policy research project commissioned by the MOHW, and the interviews were completed prior to the launch of Phase 1, the findings were used to inform recommendations for the initial HBPC model design. These recommendations were communicated to MOHW through interim and final reporting meetings and the submission of a final policy research report before the Phase 1 recruitment notice was issued in October 2022.

### Part 2. Document analysis of HBPC pilot program policies

#### Data sources and collection

We retrieved 38 documents from the official website of the Ministry of Health and Welfare using the keyword “home-based medical care.” The documents were published between October 12, 2022 (Phase 1 recruitment notice) and June 26, 2026 (latest guideline). The dataset included public notices, detailed implementation plans, operational manuals, and press releases issued by the Ministry of Health and Welfare between 2022 and 2025 (MOHW, 2022a-c, 2023a-g, 2024a-f, 2025a,b; see Panel B in Table 1; additional documents analyzed are listed in Supplementary Table 1).

#### Analysis

We conducted a directed qualitative content analysis following the READ approach: Reading materials, Extracting data, Analyzing data, and Distilling findings (Bowen, 2009; Dalglish et al., 2020). All documents were coded using Olsan’s 5P framework, with additional variables for publication date, document type, pilot phase, and a change log to track policy modifications across implementation phases (Bowen, 2009).

## Results

### Part 1. Stakeholder interviews: HBPC components by the 5P framework

The study sample (*n *= 42) comprised 66.7% male participants (Table 2). Regarding occupational roles, 38.1% were practicing physicians, 33.3% were university professors, and 28.6% were health policy researchers. Among the professors, academic affiliations were concentrated in medicine (45.8%), nursing (25.0%), and social welfare (16.7%). Analysis identified five core components of the HBPC model, aligned with the 5P framework—Purpose, Population, People, Process, and Patterns—and encompassing ten thematic domains (Table 3). These findings were synthesized into a policy report submitted to the MOHW in 2021 to inform the design of Korea’s HBPC pilot program (Kim et al., 2021)

#### Purpose

Participants agreed that the primary aims of HBPC should be to support older adults in aging at home, maintain functional ability, and reduce avoidable healthcare expenditures. To achieve these goals, stakeholders highlighted several operational priorities: improving access for older adults with mobility limitations, delivering integrated and continuous services, strengthening linkages with the Long-Term Care system, and preventing unnecessary hospitalizations and institutionalization. The provision of home-based end-of-life care also emerged as a key objective. However, participants identified several barriers to implementation, including limited workforce capacity, fragmented service delivery, and policy misalignment across sectors. These findings suggest that HBPC is not envisioned as a discrete intervention, but rather as a comprehensive, integrated response to both daily care needs and complex transitions in health status.

#### Population

The “Population” domain focused on determining who should be served by HBPC. Discussions coalesced around two major themes: identifying the appropriate target population and establishing referral pathways from community-based sources.

Participants first differentiated between short-term and long-term priorities. In the short term, they recommended prioritizing older adults classified as Levels 1–5 under Korea’s Long-Term Care eligibility rating system. These levels are assigned according to the severity of functional dependency and care needs, with Level 1 indicating the highest level of dependency. Such individuals often experience substantial functional decline and encounter difficulties accessing healthcare services. In the longer term, participants advocated for a gradual expansion of HBPC to include broader groups, such as older adults at high risk of institutionalization, individuals transitioning from Long-Term Care facilities to the community, those requiring home-based palliative care, patients recovering from acute illnesses, and individuals in need of short-term post-discharge support.

The second theme emphasized the need to build stable and consistent referral mechanisms to identify eligible recipients. Participants proposed creating coordinated networks with Long-Term Care institutions, local community service centers, hospitals involved in care transitions, and Long-Term Care hospitals to facilitate timely and appropriate referrals.

#### People

The “People” domain addressed the types of implementing institutions, governance structures, interdisciplinary workforce composition, and strategies to strengthen workforce capacity. Stakeholders expressed differing views on how HBPC teams should be organized. While all participants assumed the designation of both private and public healthcare institutions, some advocated for relaxed designation criteria to enhance scalability, whereas others preferred stricter requirements to ensure service quality. Proposed strategies included leveraging the nationwide network of public health centers and developing facility allocation plans based on regional characteristics, such as rural–urban differences. Several participants also recommended establishing a central authority to oversee national-level implementation and coordination.

Perspectives also varied regarding team composition and operational flexibility. There was broad agreement on the need for a minimum core team, comprising a physician, nurse, and social worker, with the option to include additional roles such as nurse practitioners (NPs), physical therapists, occupational therapists (OTs), and dietitians. Rather than enforcing a standardized national model, stakeholders supported flexible staffing standards tailored to regional workforce conditions, with role substitution strategies (e.g., NPs or OTs substituting in undersupplied regions) proposed to mitigate personnel shortages. To promote workforce sustainability, participants emphasized the importance of a reimbursement mechanism that provides competitive and stable compensation.

Finally, participants proposed a range of strategies for training and capacity building to enhance provider competence. Recommendations included incorporating HBPC-related content into undergraduate and graduate curricula, integrating interprofessional education into formal academic programs, and establishing accredited training modules through professional societies. Additional suggestions involved providing clinical training opportunities through university hospitals, offering continuous education within HBPC implementation sites, and fostering peer learning and community-based capacity-building programs for HBPC providers.

#### Process

The “Process” domain addressed both the direct services and supporting systems encompassed within HBPC delivery. Services were categorized into core components, which represent the minimum standards for comprehensive, continuous, and coordinated care, and enhanced components, which aim to prevent institutionalization and support high-acuity needs. Core service components included: initial patient assessment and enrollment; comprehensive geriatric assessment and individualized care planning; interdisciplinary case conferences; regular home visits for ongoing case management; family counseling and caregiver education; and coordination of community resources, including interagency collaboration. Enhanced components expanded upon these foundational services and included: 24/7 emergency consultation or home visits; proactive, home-based rehabilitation services; support for short-term inpatient admissions and discharge transitions; and home-based palliative and end-of-life care.

#### Patterns

The “Patterns” domain focused on outcome indicators for evaluating HBPC performance. Participants identified five key performance metrics: patient satisfaction, clinical effectiveness, healthcare utilization, healthcare expenditures, and the proportion of patients receiving home-based end-of-life care. Given HBPC’s multidimensional nature, stakeholders emphasized that no single indicator could adequately capture program effectiveness. Accordingly, they recommended developing a comprehensive performance management system that integrates multiple outcome measures, a central priority for future research and policy development.

### Part 2. Document analysis: final HBPC pilot model and national expansion

#### Final service delivery model and expansion trajectory

A document review of HBPC pilot program policies and implementation guidelines was conducted to examine how the program’s design and operations evolved across its three phases. The final service delivery model and the program’s national expansion from Phase 1 to Phase 4 are illustrated in Figure 1. As shown in Figure 1, Panel A, the HBPC service delivery process comprises four core stages: (1) building community-based care networks, (2) patient enrollment, (3) comprehensive assessment, and (4) integrated case management. The HBPC pilot program is designed to support healthy aging in place by delivering adequate medical and supportive services in patients’ homes, reflecting the primary stakeholder-identified goal of enabling older adults to remain in their communities for as long as possible. Eligibility is limited to community-dwelling Long-Term Care beneficiaries with substantial mobility limitations who are assessed by a physician as requiring home-based medical care. Priority enrollment is given to individuals classified as Long-Term Care Levels 1 or 2. Each care team includes one physician (general practitioner or specialist), one nurse (with either at least 24 months of clinical experience or certification as a home care advanced practice nurse), and one social worker. The HBPC model provides integrated case management, encompassing comprehensive assessments, interdisciplinary case conferences, individualized care planning, home-based team visits, and ongoing review of patient status. Services are reimbursed through a mixed-payment scheme drawing from both National Health Insurance and Long-Term Care Insurance. The pilot was launched with 28 participating sites in 2022 (Phase 1), expanded to 95 sites by 2024 (Phase 2), further increased to 344 sites in 2025 (Phase 3), and reached 463 participating sites in 2026 (Phase 4) (Figure 1, Panel B). According to the 2025 National Policy Implementation Plan, the HBPC program is expected to expand to 650 sites nationwide by 2030 (Ministry of Health and Welfare, 2025a).

**Figure 1:**
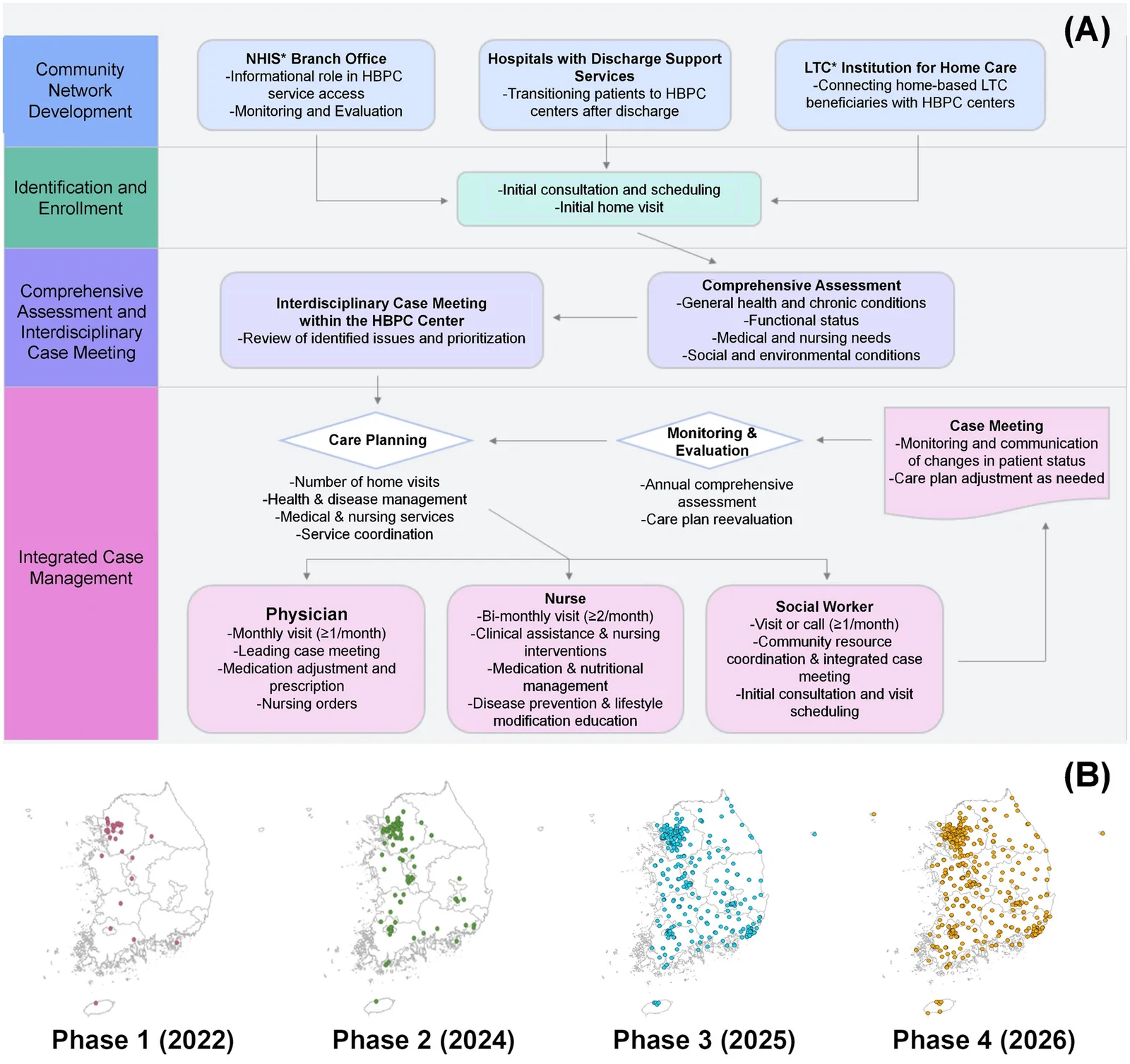
Service delivery process and implementation expansion of Korea’s National HBPC Pilot Program. HBPC = home-based primary care; LTC = long-term care; NHIS = National Health Insurance Service. (A) Service delivery process of the National HBPC Pilot Program in Korea. Adapted from the service delivery model presented in the official guidelines for Korea’s National HBPC Pilot Program (Ministry of Health and Welfare, 2024a). (B) Implementation and expansion of the HBPC pilot program across three phases.

#### Policy and operational adjustments across pilot phases

An analysis of pilot program guidelines and policy briefs revealed several key adjustments to eligibility criteria, provider scope, cost-sharing mechanisms, and operational procedures across Phases 1–4 of the HBPC pilot program, summarized in Table 4.

**Table 4:** Key changes in HBPC pilot program policy and operations across phases 1–4.

Category	Phase 1 (2022)	Phase 2 (2024)	Phase 3 (2025)	Phase 4 (2026)
**Eligible participants**	LTC levels 1–4 beneficiaries	Expanded to include LTC level 5 and cognitive support grade beneficiaries	–	–
**Eligible providers**	Institutions participating in the NHI primary care home visit pilot; public healthcare institutions (excluding tertiary hospitals); public or local health centers	–	Expanded to include local public hospitals	Further expanded to allow private hospital-level institutions in medically underserved areas
**Delivery model**	Dedicated model only (physician, nurse, and social worker within a single institution)	–	–	Clinic-public health center collaboration model introduced; One public health center may serve up to two clinics
**Monthly visit cap per physician**	100 visits/month	–	–	Increased to 140 visits/month
**Coinsurance rate^a^**	30%	–	Reduced from 30% to 15% for (1) bedridden LTC level 1–2 beneficiaries and (2) severe home-based patients using medical devices	–
**HBPC basic fee base month**	Rolling one-month interval from initial comprehensive assessment date	Shift to calendar-month basis	–	–
**Minimum time for comprehensive assessment care plan**	Not specified	Not specified	Not specified	Minimum 30 minutes required
**Annual reassessment timing**	Exactly 1 year from prior assessment date	–	Completion permitted by the end of the month following the last comprehensive assessment	–
**Provider training requirement**	Not required	Not required	Not required	Mandatory training introduced for participating physicians, nurses, and social workers

*Notes.* HBPC = home-based primary care; LTC = long-term care; NHI = National Health Insurance; “-” = same as previous phase.

a The service fee consists of the home-visit consultation, HBPC basic fee, ongoing management fee, and additional nursing fee. The coinsurance rate applies only to the home-visit consultation and additional nursing fee; the HBPC basic fee and ongoing management fee are fully covered by the NHI Service when eligibility criteria are met.

In Phase 2, participant eligibility was expanded to include Long-Term Care Level 5 beneficiaries and individuals with a cognitive support grade, a classification within Korea’s Long-Term Care Insurance system for older adults with dementia or mild cognitive impairment, where Level 1 denotes the highest level of dependency. This expansion was intended to enable older adults with dementia who face challenges visiting clinics or hospitals to receive appropriate care at home.

Eligible providers in Phases 1 and 2 included institutions participating in the National Health Insurance Primary Care Home Visit Pilot, a nationwide fee-for-service scheme reimbursing clinics for episodic physician home visits, though lacking HBPC’s interdisciplinary and longitudinal care components, along with public healthcare institutions (excluding tertiary hospitals) and local health centers. In Phase 3, a dedicated reimbursement mechanism was introduced for municipally operated local public hospitals, which are established to deliver essential medical services in underserved areas. This adjustment expanded provider participation and improved access to home-based care in medically marginalized regions.

During Phases 1 and 2, beneficiaries were required to pay a 30% coinsurance rate for eligible HBPC services, while the remaining costs were covered by the National Health Insurance Service. In Phase 3, the beneficiary coinsurance rate was reduced from 30% to 15% for two high-need populations: (1) bedridden Long-Term Care Levels 1–2 beneficiaries and (2) severely ill patients receiving home-based care with the aid of medical devices.

Additional operational changes were introduced from Phase 2 onward to improve administrative flexibility. Phase 2 replaced the rolling one-month billing cycle for HBPC reimbursement with a calendar-month cycle, allowing reimbursement eligibility to be determined according to a standard calendar month rather than an individualized monthly period based on each patient’s enrollment date. In Phase 3, the annual reassessment requirement was modified to allow completion by the end of the subsequent month, rather than requiring exact completion one year after the prior assessment. Following the implementation of the Integrated Care Support Act in March 2026, Phase 4 introduced several notable changes. Provider eligibility was further expanded to include hospital-level institutions in underserved areas, and a clinic–public health center collaboration model was newly established. The monthly physician visit cap was also raised to 140; a minimum of 30 minutes was required for the initial comprehensive assessment and care planning, and mandatory training was introduced for all participating team members.

## Discussion

This study synthesized stakeholder interviews and policy document analysis to identify design imperatives for Korea’s HBPC model and to describe how these priorities were incorporated across Pilot Phases 1–4. Stakeholder input, analyzed through the 5P framework (Purpose, Population, People, Process, and Patterns), defined priorities for service goals, target populations, team structure, delivery mechanisms, and evaluation metrics. Document analysis revealed a policy trend toward increased accessibility and financial protection, including expanded eligibility and reduced cost-sharing for high-need groups.

Although most experts supported progressive population expansion, views differed on the scope. Some favored limiting eligibility to long-term care beneficiaries, while others advocated inclusion of high-risk individuals, such as those in transitional care or requiring end-of-life services. The pilot initially targeted Long-Term Care levels 1–4, with Phase 2 extending eligibility to level 5 and cognitively impaired individuals. Future efforts should focus on identifying and reaching community-dwelling older adults in underserved regions.

Provider eligibility criteria were also contested. Some supported lower thresholds to enable scale-up, while others emphasized stricter standards to preserve care quality. The program began with institutions in the National Health Insurance Primary Care Home Visit Pilot, public healthcare centers (excluding tertiary hospitals), and local health centers. By Phase 3, eligibility was extended to municipally operated public hospitals, enhancing coverage in under-resourced areas. Further strategies are needed to position HBPC as a scalable solution for unmet needs in low-density, infrastructure-poor regions.

Document analysis indicates a clear policy shift toward promoting social equity, most notably through reduced patient cost-sharing and expanded provider eligibility to improve access in underserved areas. A recent scoping review similarly found that homebound older adults face substantial care gaps, including unmet dental, nutritional, and preventive needs, along with heightened risks of falls, functional decline, and social isolation (McManus et al., 2025). These findings reinforce the need for integrated, home-based care models that address both medical and social vulnerabilities. Evidence from the U.S. Independence at Home demonstration supports this perspective: in Year 9, all estimated savings occurred among Medicare–Medicaid dually eligible beneficiaries, primarily due to reduced inpatient and skilled nursing facility use (CMS, 2025). This suggests that HBPC may improve care continuity and quality for vulnerable populations, thereby reducing reliance on costly institutional care. For Korea’s HBPC model, future research should identify which subgroups benefit most and ensure that policy refinements continue to prioritize access for socioeconomically and medically vulnerable older adults.

This study provides early evidence to guide real-world policy decisions on launching and refining Korea’s HBPC pilot program. While integrating stakeholder interviews and policy documents offered a comprehensive view of design priorities and their uptake, further development is needed, particularly within the “Patterns” domain of the 5P framework. Expert perspectives on success metrics, including quality, cost, and patient satisfaction, remain preliminary. Document analysis also revealed underdeveloped systems for interdisciplinary team training and performance evaluation, both of which are key areas for future policy improvement. A formal evaluation system for assessing service quality and goal achievement remains under development. Beginning in 2025, the MOHW commissioned policy research to develop quality assessment and management indicators for HBPC centers. Further evaluation studies are needed to examine the pilot program’s effectiveness.

A notable limitation is the absence of service user input, such as from older adults and family caregivers; incorporating these perspectives is essential for a comprehensive patient-centered evaluation. Future research should engage a broader stakeholder base and establish validated performance indicators to support a robust evaluation framework. Despite these limitations, the study offers timely insights into the foundational design and policy trajectory of Korea’s HBPC model, with implications for its sustainable scaling. Korea’s experience may also inform other rapidly aging health systems aiming to integrate medical and long-term care while advancing equity.

## Conclusion

This study offers a structured foundation for developing Korea’s HBPC model, drawing on stakeholder interviews and policy document analysis. It underscores the value of incorporating stakeholder perspectives to ensure that model design reflects frontline expertise. Policy changes from Phase 1 to Phase 4 broadened eligibility, expanded provider participation, and reduced cost-sharing for high-need patients, measures that improve service accessibility. These findings highlight HBPC’s role in supporting aging in place, especially for high-need and vulnerable populations, and provide guidance for building responsive, inclusive models of community-based care.

## Supplementary Material

igag084_Supplementary_Data

## Data Availability

The original data are not available for secondary analysis in order to protect participant confidentiality. The study was not preregistered.
