# Supplementary material for: From design to implementation: policy evolution of home-based primary care in Korea
Source: Innov Aging. 2026 Jul 21;10(10):igag084. doi: 10.1093/geroni/igag084 (PMC13626712; doi:10.1093/geroni/igag084)
Supplement: Supplementary file 1 [file igag084_supplementary_data.pdf]

***Innovation in Aging* Supplementary Material: Choi et al. From design to implementation: Policy evolution of home-based primary care in Korea.**

Supplementary Table 1. List of documents included in the HBPC pilot program policy review

No.	Document title	Document type	Phase of pilot program	Date posted on website	Purpose	Ref
1	Announcement on Designation of Home-Based Primary Care Centers for the Pilot Program	Public notice	Phase 1	October 12, 2022	Recruit participating institutions and announce details for Phase 1 of the pilot	MOHW*, 2022a
2	Announcement on Home-Based Primary Care Center Pilot Program	Public notice	Phase 2	November 15, 2023	Recruit participating institutions and announce details for Phase 2 of the pilot	MOHW, 2023a
3	Additional Selection Notice for the 2nd Phase of the Home-Based Primary Care Pilot Program	Public notice	Phase 2	January 12, 2024	Recruit additional participating institutions for Phase 2 of the pilot	MOHW, 2024b
4	Announcement on Long-Term Care Home-Based Primary Care Center Pilot Program	Public notice	Phase 3	November 12, 2024	Recruit participating institutions and announce details for Phase 3 of the pilot.	MOHW, 2024c
5	Final Detailed Implementation Plan for the Home-Based Primary Car Center Pilot Project	Implementation plan	Phase 1	October 12, 2022	Outline operational details and participation rules for Phase 1	MOHW, 2022a
6	Implementation Plan for the Home-Based Primary Care Center Pilot Program	Implementation plan	Phase 2	November 15, 2023	Outline operational details and participation rules for Phase 2	MOHW, 2023a
7	Detailed Implementation Plan for the Long-Term Care Home-Based Primary Center Pilot Project	Implementation plan	Phase 3	November 12, 2024	Outline operational details and participation rules for Phase 3	MOHW, 2024c
8	Guidelines for the Long-Term Care Home-Based Primary Care Center Pilot Program	Guidelines	Phase 1	December 2, 2022	Provide detailed operational guidance for Phase 1 of the pilot	MOHW, 2022b
9	Draft Revision of the Guidelines for the Long-Term Care Home-Based Primary Care Center Pilot Program	Guidelines	Phase 1	June 1, 2023	Present proposed updates to Phase 1 guidelines	MOHW, 2023b
10	Final Guidelines for the Long-Term Care Home-Based Primary Care Center Pilot Program	Guidelines	Phase 2	January 8, 2024	Provide detailed operational guidance for Phase 2 of the pilot	MOHW, 2024a
11	Overview of the Long-Term Care Home-Based Primary Care Center Pilot Program	Guidelines	Phase 2	January 12, 2024	Summarize the purpose, scope, and main procedures for Phase 2 of the pilot	MOHW, 2024b
12	Comparative Table of Old and New Provisions – Standards for	Guidelines	Phase 3	January 8, 2025	Highlight and compare key Phase 3 guideline revisions	MOHW, 2025b

No.	Document title	Document type	Phase of pilot program	Date posted on website	Purpose	Ref
	Clinics and Local Public Hospitals, Key Revisions to the Home-Based Primary Care Pilot Program Guidelines				against previous versions	
13	Guidelines for the Long-Term Care Home-Based Primary Care Pilot Program (Clinics and Local Public Hospitals)	Guidelines	Phase 3	January 8, 2025	Provide Phase 3 operational guidance for clinics and local public hospitals	MOHW, 2025b
14	Guidelines for the Long-Term Care Home-Based Primary Care Pilot Program (Local Public Health Institutions)	Guidelines	Phase 3	January 8, 2025	Provide Phase 3 operational guidance for local public health institutions	MOHW, 2025b
15	Guidelines for the Long-Term Care Home-Based Primary Care Pilot Program (Oriental Medicine Clinics)	Guidelines	Phase 3	January 8, 2025	Provide Phase 3 operational guidance for oriental medicine clinics	MOHW, 2025b
16	Guidelines for the Long-Term Care Home-Based Primary Care Pilot Program (Oriental Medicine Clinics)	Guidelines	Phase 3	January 8, 2025	Provide annex forms and templates for Phase 3 implementation	MOHW, 2025b
17	Morning Press Release (Oct 12): Home-Based Primary Care Centers Taking a Leap Forward to Reach Mobility-Impaired Older Adults	Press release	Phase 1	October 11, 2022	Highlight the launch of HBPC centers serving mobility-impaired older adults	MOHW, 2022c
18	Morning Press Release (Feb 8): 28 Long-Term Care Home-Based Primary Care Centers Officially Launch	Press release	Phase 1	February 7, 2023	Announce the official start of 28 LTC HBPC centers	MOHW, 2023c
19	Press Reference Material: Ministry of Health and Welfare Listens to On-Site Challenges of Home-Based Primary Care Centers	Press release	Phase 1	April 21, 2023	Communicate ministry engagement with on-site operational challenges	MOHW, 2023d
20	Press Reference Material: Visiting Home-Based Primary Care Centers Serving Mobility-Impaired Older Adults to Hear Voices from the Field	Press release	Phase 1	May 12, 2023	Report ministry visits to HBPC centers to hear feedback from the field	MOHW, 2023e
21	Press Reference Material: Ministry of Health and Welfare Inspects Field Services Ahead of Next Year's Expansion of the Home-Based Primary Care Pilot Program	Press release	Phase 1	November 10, 2023	Announce field inspections ahead of next year's pilot program expansion	MOHW, 2023f

No.	Document title	Document type	Phase of pilot program	Date posted on website	Purpose	Ref
22	Morning Press Release (Nov 15): Expanded Home Medical Services for Mobility-Impaired Older Adults through the Long-Term Care Home-Based Primary Care Centers	Press release	Phase 1	November 14, 2023	Highlight expansion of home medical services for mobility-impaired older adults	MOHW, 2023g
23	Morning Press Release (Jan 5): Second Phase of the Long-Term Care Home-Based Primary Care Center Pilot Program Launched with 83 Centers	Press release	Phase 2	January 4, 2024	Announce launch of Phase 2 with 83 participating HBPC centers	MOHW, 2024d
24	Morning Press Release (Mar 6): Long-Term Care Beneficiaries to Access Home Medical Services in 72 Cities and Counties Starting March	Press release	Phase 2	March 5, 2024	Inform beneficiaries of service availability across 72 municipalities starting March	MOHW, 2024e
25	Morning Press Release (Nov 12): Recruiting Local Governments and Medical Institutions to Join the Long-Term Care Home-Based Primary Care Pilot Program	Press release	Phase 2	November 11, 2024	Recruit local governments and medical institutions for Phase 3 participation	MOHW, 2024f
26	Morning Press Release (Dec 20): Long-Term Care Home-Based Primary Care Centers to Provide Services Nationwide from 135 Locations Starting January 2025	Press release	Phase 2	December 19, 2024	Announce nationwide rollout of HBPC services from 135 centers in January 2025	MOHW, 2024g
27	Announcement of Additional Recruitment for the Long-Term Care Home-Based Primary Care Center Pilot Program	Public notice	Phase 3	May 19, 2025	Recruit additional participating institutions for Phase 3 of the pilot	MOHW, 2025c
28	Overview of the Long-Term Care Home-Based Primary Care Center Pilot Program	Guidelines	Phase 3	May 19, 2025	Summarize the purpose, scope, and main procedures for Phase 3 of the pilot	MOHW, 2025d
29	Announcement of Recruitment for Participating Institutions in the Long-Term Care Home-Based Primary Care Center Pilot Program	Public notice	Phase 4	October 28, 2025	Recruit participating institutions and announce details for Phase 4 of the pilot	MOHW, 2025e
30	First Round Recruitment Announcement for the 2026 Long-Term Care Home-Based Primary Care Center Pilot Program	Public notice	Phase 4	January 6, 2026	Recruit additional participating institutions for Phase 4 of the pilot	MOHW, 2026a
31	Guidelines for the Long-Term	Guidelines	Phase 4	February 4,	Provide Phase 4	MOHW,

No.	Document title	Document type	Phase of pilot program	Date posted on website	Purpose	Ref
	Care Home-Based Primary Care Center Pilot Program (Clinics, Hospitals, and Local Public Health Institutions – Western Medicine)			2026	operational guidance for clinics, hospitals, and local public health institutions (Western medicine)	2026b
32	Guidelines for the Long-Term Care Home-Based Primary Care Center Pilot Program (Clinic–Public Health Center Collaboration Model – Western Medicine)	Guidelines	Phase 4	February 4, 2026	Provide Phase 4 operational guidance for the collaboration model (Western medicine)	MOHW, 2026c
33	Guidelines for the Long-Term Care Home-Based Primary Care Center Pilot Program (Clinic–Public Health Center Collaboration Model – Korean Medicine)	Guidelines	Phase 4	February 4, 2026	Provide Phase 4 operational guidance for the collaboration model (Korean medicine)	MOHW, 2026d
34	Guidelines for the Long-Term Care Home-Based Primary Care Center Pilot Program (Korean Medicine Clinics and Local Public Health Institutions)	Guidelines	Phase 4	February 4, 2026	Provide Phase 4 operational guidance for Korean medicine clinics and local public health institutions	MOHW, 2026e
35	Comparative Table of Key Revisions to the Long-Term Care Home-Based Primary Care Pilot Program Guidelines (Clinics, Hospitals, and Local Public Health Institutions – Western Medicine)	Guidelines	Phase 4	February 4, 2026	Highlight and compare key Phase 4 guideline revisions (Western medicine) against previous versions	MOHW, 2026f
36	Comparative Table of Key Revisions to the Long-Term Care Home-Based Primary Care Pilot Program Guidelines (Korean Medicine Clinics and Local Public Health Institutions)	Guidelines	Phase 4	February 4, 2026	Highlight and compare key Phase 4 guideline revisions (Korean medicine) against previous versions	MOHW, 2026g
37	Second Round Additional Recruitment Announcement for the 2026 Long-Term Care Home-Based Primary Care Center Pilot Program	Public notice	Phase 4	April 21, 2026	Recruit additional participating institutions for Phase 4 of the pilot (second round)	MOHW, 2026h
38	Standards for Medically Underserved Areas (Emergency, Income Tax Act, and Delivery Services)	Guidelines	Phase 4	April 21, 2026	Define criteria for medically underserved area designation used in Phase 4 eligibility determination	MOHW, 2026i

Notes. MOHW = Ministry of Health and Welfare; HBPC = home-based primary care; LTC = long-term care.

References: Additional policy documents

Ministry of Health and Welfare. (2024g, December 19). From January 2025, Home Medical Center Services to be Provided at 135 Long-Term Care Home Medical Centers Nationwide. https://www.mohw.go.kr/board.es?mid=a10503010100&bid=0027&act=view&list_no=1483985

Ministry of Health and Welfare. (2025c, May 19). Announcement of additional recruitment for the long-term care home-based primary care center pilot program. https://www.mohw.go.kr/board.es?mid=a10501010100&bid=0003&act=view&list_no=1486009&tag=&nPage=1

Ministry of Health and Welfare. (2025d, May 19). Overview of the long-term care home-based primary care center pilot program. https://www.mohw.go.kr/board.es?mid=a10501010100&bid=0003&act=view&list_no=1486009&tag=&nPage=1

Ministry of Health and Welfare. (2025e, October 28). Announcement of recruitment for participating institutions in the long-term care home-based primary care center pilot program. https://www.mohw.go.kr/board.es?mid=a10501010100&bid=0003&act=view&list_no=1487716&tag=&nPage=1

Ministry of Health and Welfare. (2026a, January 6). First round recruitment announcement for the 2026 long-term care home-based primary care center pilot program. https://www.mohw.go.kr/board.es?mid=a10501010100&bid=0003&act=view&list_no=1488521&tag=&nPage=1

Ministry of Health and Welfare. (2026b, February 4). Guidelines for the long-term care home-based primary care center pilot program (clinics, hospitals, and local public health institutions – Western medicine). https://www.mohw.go.kr/board.es?mid=a10501010100&bid=0003&act=view&list_no=1488980&tag=&nPage=1

Ministry of Health and Welfare. (2026c, February 4). Guidelines for the long-term care home-based primary care center pilot program (clinic–public health center collaboration model – Western medicine). https://www.mohw.go.kr/board.es?mid=a10501010100&bid=0003&act=view&list_no=1488980&tag=&nPage=1

Ministry of Health and Welfare. (2026d, February 4). Guidelines for the long-term care home-based primary care center pilot program (clinic–public health center collaboration model – Korean medicine). https://www.mohw.go.kr/board.es?mid=a10501010100&bid=0003&act=view&list_no=1488980&tag=&nPage=1

Ministry of Health and Welfare. (2026e, February 4). Guidelines for the long-term care home-based primary care center pilot program (Korean medicine clinics and local public health

institutions). https://www.mohw.go.kr/board.es?mid=a10501010100&bid=0003&act=view&list_no=1488980&tag=&nPage=1

Ministry of Health and Welfare. (2026f, February 4). Comparative table of key revisions to the long-term care home-based primary care pilot program guidelines (clinics, hospitals, and local public health institutions – Western medicine). https://www.mohw.go.kr/board.es?mid=a10501010100&bid=0003&act=view&list_no=1488980&tag=&nPage=1

Ministry of Health and Welfare. (2026g, February 4). Comparative table of key revisions to the long-term care home-based primary care pilot program guidelines (Korean medicine clinics and local public health institutions). https://www.mohw.go.kr/board.es?mid=a10501010100&bid=0003&act=view&list_no=1488980&tag=&nPage=1

Ministry of Health and Welfare. (2026h, April 21). Second round additional recruitment announcement for the 2026 long-term care home-based primary care center pilot program. https://www.mohw.go.kr/board.es?mid=a10501010100&bid=0003&act=view&list_no=1490198&tag=&nPage=1

Ministry of Health and Welfare. (2026i, April 21). Standards for medically underserved areas (emergency, income tax act, and delivery services). https://www.mohw.go.kr/board.es?mid=a10501010100&bid=0003&act=view&list_no=1490198&tag=&nPage=1
